# The Effects of Surgical Antiseptics and Time Delays on RNA Isolated From Human and Rodent Peripheral Nerves

**DOI:** 10.3389/fncel.2019.00189

**Published:** 2019-05-22

**Authors:** Matthew Wilcox, Tom J. Quick, James B. Phillips

**Affiliations:** ^1^Peripheral Nerve Injury Research Unit, Royal National Orthopaedic Hospital, Stanmore, United Kingdom; ^2^Department of Pharmacology, UCL School of Pharmacy, University College London, London, United Kingdom; ^3^UCL Centre for Nerve Engineering, University College London, London, United Kingdom

**Keywords:** peripheral nerve regeneration/repair, RNA isolation and purification, RT-qPCR, RNA seq, surgery, cellular and molecular biology, human tissue, surgical antisepsis

## Abstract

Peripheral Nerve Injury (PNI) is common following blunt or penetrating trauma with an estimated prevalence of 2% among the trauma population. The resulting economic and societal impacts are significant. Nerve regeneration is a key biological process in those recovering from neural trauma. Real Time-quantitative Polymerase Chain Reaction (RT-qPCR) and RNA sequencing (RNA seq) are investigative methods that are often deployed by researchers to characterize the cellular and molecular mechanisms that underpin this process. However, the ethical and practical challenges associated with studying human nerve injury have meant that studies of nerve injury have largely been limited to rodent models of renervation. In some circumstances it is possible to liberate human nerve tissue for study, for example during reconstructive nerve repair. This complex surgical environment affords numerous challenges for optimizing the yield of RNA in sufficient quantity and quality for downstream RT-qPCR and/or RNA seq applications. This study characterized the effect of: (1) Time delays between surgical liberation and cryopreservation and (2) contact with antiseptic surgical reagents, on the quantity and quality of RNA isolated from human and rodent nerve samples. It was found that time delays of greater than 3 min between surgical liberation and cryopreservation of human nerve samples significantly decreased RNA concentrations to be sub-optimal for downstream RT-qPCR/RNA seq applications (<5 ng/μl). Minimizing the exposure of human nerve samples to antiseptic surgical reagents significantly increased yield of RNA isolated from samples. The detrimental effect of antiseptic reagents on RNA yield was further confirmed in a rodent model where RNA yield was 8.3-fold lower compared to non-exposed samples. In summary, this study has shown that changes to the surgical tissue collection protocol can have significant effects on the yield of RNA isolated from nerve samples. This will enable the optimisation of protocols in future studies, facilitating characterisation of the cellular and molecular mechanisms that underpin the regenerative capacity of the human peripheral nervous system.

## Introduction

Peripheral nerve injury (PNI) is a common outcome following blunt or penetrating trauma with an estimated prevalence of 2% among the trauma population (Brattain, 2013). The resulting economic and societal ramifications are significant (Taylor et al., 2008). Nerve regeneration is a key biological process in those recovering from neural trauma. The cellular and molecular mechanisms that underpin this process have been well characterized in rodent models of PNI and have been central to therapeutic advancements made in this field of regenerative neuroscience (Kaplan et al., 2015; Jessen and Mirsky, 2016). Investigative methods such as RNA seq (RNA sequencing) and/or Real Time-quantitative Polymerase Chain Reaction (RT-qPCR) are often deployed in studies to characterize these mechanisms (Bosse et al., 2001; Jiang et al., 2014; Clements et al., 2017; Yi et al., 2017). However, the ethical and practical challenges associated with studying human nerve injury have meant that studies of nerve injury have largely been limited to rodent models of damage and renervation. For example, it is challenging to liberate human nerve samples without worsening patient morbidity. Moreover, traumatic nerve injuries represent a highly heterogeneous cohort and a standardized model in which human nerve regeneration can be studied is awaited.

In some circumstances it is possible to liberate human nerve tissue for study, for example during reconstructive nerve repair. Samples that can be extracted are often finite and are exposed to the complex surgical environment, which includes chemical and physical environmental factors, time pressures and other priorities which are not present when sampling animal tissues in a laboratory setting. This affords numerous challenges when optimizing protocols for the extraction of RNA with sufficient quantity and quality for quantitative analysis, therefore this study is dedicated to exploring these challenges.

The extraction of RNA in sufficient quantity and quality is a critical step toward obtaining valid RT-qPCR/RNA seq results (Atz et al., 2007; Popova et al., 2008; Abasolo et al., 2011). A minimum concentration of 5 ng/μl is often used for the synthesis of single stranded complementary DNA (cDNA) (Fox et al., 2012; França et al., 2012) and in the quantitative and qualitative assessments of RNA yields; a critical step toward valid RT-qPCR and/or RNA seq results (Bastard et al., 2002; Fleige and Pfaffl, 2006; Wilkes et al., 2010). The quality of RNA can be determined using quantitative and qualitative assays; 260/280 ratios and electropherograms respectively (Mee et al., 2011; Yockteng et al., 2013; Samadani et al., 2015; Walker et al., 2016). When optimizing RNA extraction protocols to attain yields sufficient for RT-qPCR assays, it is necessary to consider the tissue that is being processed; a review of the literature highlights differentials in the yield of RNA isolated from different tissues (Ruettger et al., 2010; Walker et al., 2016; Grinstein et al., 2018).

Based on experiences within our research unit and others, there appears to be a differential between the RNA extraction ratio [mean total RNA (μg) divided by initial tissue sample mass (mg)] of healthy and denervated nerve liberated from rats. Typical values range from 0.09 μg/mg for healthy sciatic nerve and 0.27 μg/mg for denervated sciatic nerve (Yamamoto et al., 2012; Weng et al., 2018). This differential is perhaps attributable to the presence of higher numbers of proliferating cells in denervated tissue. Moreover, the degradation of connective tissue during Wallerian degeneration is likely to make denervated tissue more amenable to complete lysis. In comparing peripheral nerve to other tissues, the reported RNA extraction ratios are considerably lower than those reported for RNA isolated from other tissues such as liver, kidney and spleen which have mean extraction ratios of 1.56 μg/mg, 0.50 μg/mg, and 0.41 μg/mg respectively (Yamamoto et al., 2012). The lower RNA yields reported from nerve samples are at least partially attributable to the fact that nerves are invested by fibrous connective tissue particularly in the epineurium (Thomas, 1963). The biomechanical properties of this tissue are antagonistic to total cellular disruption and lysis of nerve tissue which is an imperative step in RNA isolation (Bastard et al., 2002; Guan and Yang, 2008). This is an indication for the application of specialist RNA extraction kits which include a broad spectrum serine protease such as Proteinase K to facilitate optimal digestion of tissue and lysis of cells (Yockteng et al., 2013; Peeters et al., 2016; Amini et al., 2017).

Accepting that the concentration of RNA that can be isolated from peripheral nerve tissue is likely to be lower than other tissues, it is pertinent to optimize surgical protocols in order to conserve whatever RNA is available. One variable that has been shown to be predictive of the quality and quantity of RNA extracted from samples is the time interval between sample liberation and cryopreservation (Borgan et al., 2011; Hatzis et al., 2011; Caboux et al., 2013); a variable that is difficult to control in the surgical environment due to intra-operative priorities and handling limitations. It has been shown that delays of hours between sample liberation and cryopreservation impairs RNA isolated from cancerous samples (Borgan et al., 2011; Hatzis et al., 2011; Caboux et al., 2013). However, corresponding time frames using human nerve samples have not been reported.

While a number of past studies of other surgically liberated tissues for qPCR analysis have been optimized by manipulating variables such as time delays and RNA extraction protocols (Berglund et al., 2007; Borgan et al., 2011; Hatzis et al., 2011; Caboux et al., 2013), the exploration of other peri-operative variables that could impact on RNA yields, such as the chemical environment, have not been reported. The liberal application of antiseptic compounds in a surgical setting may influence RNA yields although this has not been reported previously. The most common constituents of these reagents globally are chlorhexidine and iodine (Hirsch et al., 2010). Despite *in vitro* experimental evidence demonstrating chlorhexidine and iodine based surgical antiseptic reagents can be cytotoxic to human SH-SY5Y neuroblastoma cells and rat RSC96 Schwann cell populations (Doan et al., 2012), their effects on RNA yield and quality have not been well characterized.

Protocols that detail how human nerve samples should be handled to optimize RNA yields for subsequent RT-qPCR and RNA seq analysis are not documented. This study aimed to explore the time course of RNA degradation in nerve tissue in order to establish an ideal time frame for the liberation of human nerve samples and cryopreservation (snap-freezing in liquid nitrogen). Additionally, this study aimed to investigate for the first time the effect of exposure of human nerve samples to surgical antiseptic reagents.

## Materials and Methods

### Optimizing the Human Surgical Environment for RNA Isolation

A total of 12 denervated human nerve samples were harvested from 12 different patients who underwent reconstructive surgery at the Peripheral Nerve Injury Unit, Royal National Orthopaedic Hospital after informed consent for the therapeutic procedure and for tissue donation (Table 1). Informed consent was obtained using the guidelines detailed in the UK Human Tissue Act (Human Tissue Act, 2004). Ethical approval for this project was provided by the UCL Biobank Research Committee (REC 15.15).

**Table 1:** Demographic of patients and samples included in this study.

Mechanism of injury	Intra-operative findings	Details of surgery	Nerve assessed	Denervated/ Innervated	Approximate time delay between surgical liberation and cryopreservation (min)
**Experimental group 1**					
Motorbike accident	Axonotmesis of the tibial nerve	Below the knee amputation	Tibial	Denervated	3
Motorbike accident	Right C5/6 Avulsion	Oberlin’s nerve transfer	Biceps branch of musculocutaneous	Denervated	3
Fall on to sharp cast iron railing	Axonotmesis of superficial peroneal nerve	Excision of nerve	Superficial common peroneal nerve	Denervated	3
Motorbike accident	Left C6-T1 root avulsion	Somsak’s nerve transfer	Medial head of triceps branch of the radial nerve	Denervated	3
**Experimental group 2**					
Iatrogenic nerve injury secondary to humeral fracture repair	Neurotmesis of the axillary nerve	Somsak’s nerve transfer	Axillary	Denervated	5
Car v Tree	Right C4 - T1 avulsion	Intercostal nerve transfer to musculocutaneous nerve	Biceps branch of musculocutaneous	Denervated	10
Motorbike accident	Right C5/6 Avulsion	Oberlin’s nerve transfer	Biceps branch of musculocutaneous	Denervated	15
Car v Lorry	Axonotmesis of the accessory nerve	Fascicle of C7 transfer to accessory nerve	Fascicle of C7 to pectoralis muscles to accessory nerve	Denervated	15
Motorbike accident	C5/6/7 Avulsion	Double Oberlin’s nerve transfer	Biceps branch of musculocutaneous	Denervated	20
**Experimental group 3**					
Iatrogenic nerve injury secondary to left neck lymph node biopsy	Neurotmesis of the spinal accessory nerve	Supraclavicular nerve transfer to spinal accessory	Supraclavicular and Spinal accessory	Supraclavicular (innervated) and Spinal accessory (denervated)	3
Trampoline accident	Neurotmesis of the ulnar nerve	Sural nerve autograft to ulnar	Ulnar and Sural	Ulnar (denervated), Sural (innervated)	3
Moped v Lampost	C5-8 Avulsion	Intercostal nerve transfer to triceps division of radial nerve	Radial and Intercostal	Intercostal (innervated), Radial (denervated)	3

In all cases the site of operation was prepared with chlorhexidine or iodine based antiseptic reagents in concordance with standard surgical protocol (Digison, 2007). The demographic of the study subjects and details of the nerve samples harvested are documented in Table 1.

Samples harvested were often heterogeneous in size, morphology and innervation, so they were dissected into sections measuring 0.5 ± 0.2 cm in the longitudinal orientation. The dimensions of the samples were chosen to allow comparisons with other RT-qPCR studies of rodent nerve samples which used similar dimensions (Jiang et al., 2014). Additionally, samples were characterized as denervated using intra-operative neurophysiological monitoring to record compound nerve action potentials (CNAPs) (Slimp, 2000; Herrera-Perez et al., 2015). The nerve was assumed to be denervated if a CNAP was absent and no muscle twitch was observed. Innervated samples were harvested from sites external to the site of injury (sural, intercostal and supraclavicular nerve samples) for the purpose of surgical nerve repair.

Nerve samples were stratified into 3 experimental groups (shown in Table 1):

Group 1: Samples whereby the time between sample liberation and cryopreservation was less than 3 min.
Group 2: Samples where the time interval between surgical liberation of the nerve sample and cryopreservation was greater than 3 min ranging up to 20 min.
  Since RNA yields from nerve samples remained lower than that reported in rodent studies following optimisation of handling times, the exploration of other peri-operative variables was necessitated. This informed the development of a third experimental group to explore the effect of minimizing the exposure of nerve samples to antiseptic reagents.
Group 3: Samples liberated and cryopreserved within 3 min but utilizing a “clean change” of surgical gloves and surgical equipment for harvest and handling of the sample to minimize exposure to antiseptic reagents. This group included healthy nerve samples in addition to denervated nerves.

### Isolating the Effects of Antiseptic Reagents on RNA Yield Using a Rodent Model of Peripheral Nerve Liberation

Standard international operating protocols dictate that iodine and/or chlorhexidine based antiseptic reagents should be used to prepare the site of surgical incision as detailed by the World Health Organization (WHO Surgical Site Infection Prevention Guidelines, 2016). In order to investigate the effects of these reagents on RNA, a rodent model of surgical nerve liberation was utilized in an environment that was otherwise absent of antiseptic reagents. All animal use was performed according to the UK Animal Scientific Procedures Act 1986 / the European Communities Council Directives (86/609/EEC) and approved by the UCL Animal Welfare and Ethics Review Board. A total of 9 Sprague Dawley rats (6 female and 3 male) were culled using CO_2_ inhalation and had their sciatic nerves excised. The nerves were then sharply dissected into 0.5 cm sections (to reflect the size of samples harvested from human patients). The sections were then randomized into 2 groups:

**Control group:** Samples were processed before any of the experimental samples to minimize the risk of contamination of the experimental environment.

**Experimental group:** 100 μl of one of the following commonly used surgical antiseptic reagents were applied by pipette: 10% iodine/water, 10% iodine/EtOH (Ethanol) or 2% chlorhexidine gluconate. Within 30 s of the nerve samples being excised, the nerve sample and antiseptic reagent was allowed to stand for 30 s and then immediately snap-frozen.

### RNA Extraction Protocol

All materials used in this process of RNA isolation were treated with RNase Zap (Invitrogen). Rodent and human nerves were placed into a 5 ml tube and snap frozen in liquid nitrogen. The time between tissue isolation and freezing was monitored as well as the interaction of samples with antiseptic surgical reagents. RNA was isolated from all nerve samples using the Qiagen RNeasy^®^ Fibrous Tissue Mini Kit. The total volume of eluted RNA for each sample was 40 μl.

The quantity of RNA was determined using a Tecan^TM^ Infinite 200 PRO multimode reader. Quality of RNA was measured using a NanoDrop^TM^ spectrophotometer to ascertain 260/280 ratios for each sample. Samples were also analyzed using Bio-rad Experion^TM^ RNA analysis kits to assess Ribosomal Integrity Number (RIN), and obtain electropherogram data and automated agarose gel readings from samples using the Experion^TM^ Automated Electrophoresis System.

## Results

### Optimisation of the Surgical Environment for RNA Extraction

**FIGURE 1:**
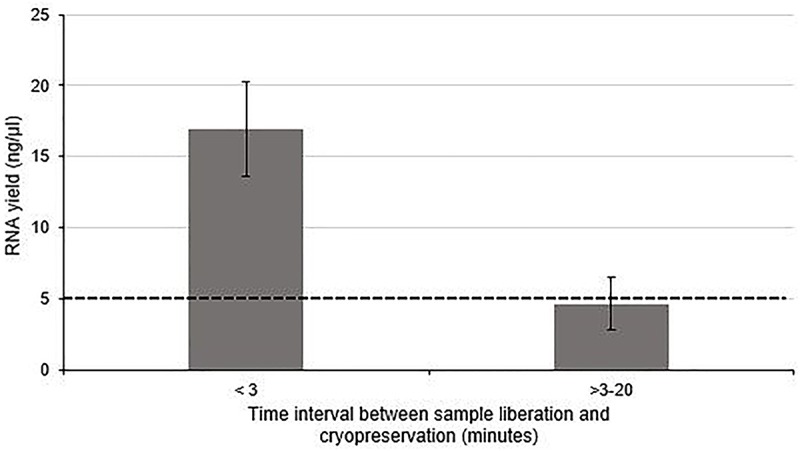
The effect of time between tissue liberation and cryopreservation on RNA yield in human nerve tissue in a surgical environment utilizing standard antiseptic protocols. The duration between nerve tissue removal and freezing was monitored and samples were grouped according to whether the delay was more than (*n* = 5) or less than 3 min (*n* = 4). There was a statistically significant difference between each group (*p* < 0 01 two tailed *t*-test). The dotted black line represents the minimum concentration of RNA often cited (Bastard et al., 2002; Fleige and Pfaffl, 2006; Wilkes et al., 2010; Fox et al., 2012; França et al., 2012) required for downstream RT-qPCR/RNA seq Data is presented as a mean ± 1 Standard Deviation (SD).

The effect of time between tissue extraction and freezing on the yield of RNA isolated from human nerve samples was investigated (Group 1 and Group 2). Figure 1 demonstrates that the optimal yield of RNA isolated from samples cryopreserved within 3 min of surgical liberation (Group 1) is approximately 3.6-fold higher than that from samples frozen after more than 3 min (Group 2) (*p* < 0.01). Importantly, the latter group had an RNA yield approximately equivalent to the minimum threshold value required for acceptable analysis and cDNA synthesis (Bastard et al., 2002; Fleige and Pfaffl, 2006; Wilkes et al., 2010; Fox et al., 2012; França et al., 2012).

**FIGURE 2:**
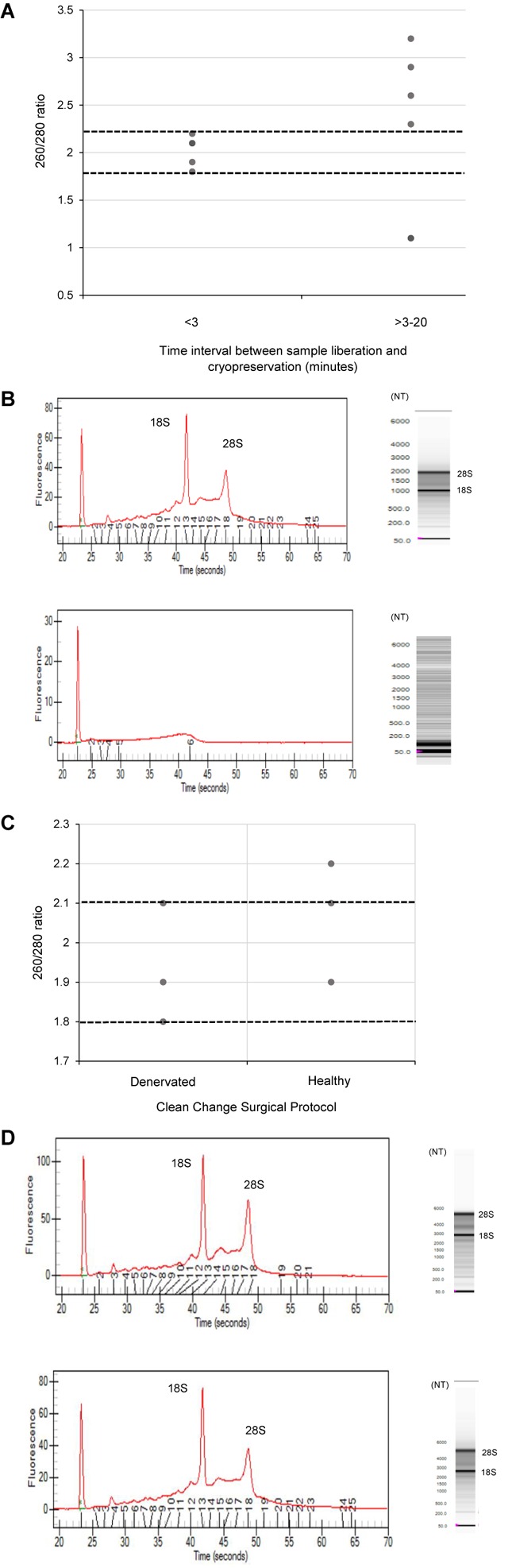
The effect of time delays and surgical antiseptic reagents on the quality of RNA isolated from human nerve tissue. **(A)** A scatter plot to demonstrate the distribution of 260/280 ratios yielded from RNA isolated from denervated human nerve samples surgically liberated and cryopreserved within 3 min (experimental Group 1) compared to those that were not cryopreserved within this timeframe (experimental Group 2). The two dotted black lines represent the range of 260/280 ratios that is predictive of high quality RNA (1.8–2.2). **(B)** Electropherograms (left) and agarose gels (right) digitally produced by the Experon^TM^Automated Electrophoresis System to assess quality of RNA isolated from denervated human nerve samples. The electropherogram is displayed with fluorescence on the *y*-axis and time of the fragment on the *x*-axis. The upper electropherogram/agrose gel represents a denervated sample cryopreserved within 3 min (Group 1) and the lower electropherogram/agrose gel represents a denervated sample that was exposed to a time delay of 20 min (Group 2). **(C)** A scatter plot to represent the 260/280 ratios yielded from healthy and denervate samples liberated under a “clean change” surgical protocol (Group 3). **(D)** Electropherogram (left) and agarose gels (right) to assess the quality of RNA isolated from healthy and denervated samples liberated under a “clean change” surgical protocol. The upper electropherogram/agarose gel represents a denervated sample (Group 3) and the lower electropherogram/agarose gel represents sural nerve (Group 3). All samples that yielded 260/280 ratios of between 1.8 and 2.2 were assessed to have Ribosomal Integrity Numbers (RIN) of between 7 and 10 (predictive of high quality RNA).

The quality of RNA extracted from these nerve samples was concurrently determined quantitatively using 260/280 absorbance ratios (Figure 2) and semi-quantitatively by analyzing the ratio of ribosomal RNA bands in agarose gels and changes in electropherogram morphology (Figure 2). Nucleic acids have an absorbance maxima at 260 nm. The ratio of this to the absorbance at 280 nm is used to determine the purity of DNA and RNA. A ratio of 1.8–2.2 is predictive of high quality RNA (Desjardins and Conklin, 2010). The distribution of 260/280 ratios assessed for samples cryopreserved within 3 min (Group 1) is shown in Figure 2, all of which fall within the optimal range (1.8–2.2). In addition, two distinct ribosomal RNA bands at 28S and 18S with a ratio of around 2.0 can be seen in the agarose gels (Figure 2) indicating high quality RNA isolated from samples processed within 3 min. Figure 2 demonstrates how this ratio is lost in samples exposed to time delays of up to 20 min (Group 2 samples). An electropherogram was also assessed to illustrate the overall size of the ribosomal peaks and to further characterize the differential in RNA quality between samples processed within 3 min and those exposed to time delays (Figure 2).

Using the data from the electropherogram reports, a RIN ranging from 1 to 10 (with 10 being predictive of high quality RNA) was assigned to each sample. RIN is generated using an algorithm that selects features from the electropherograms and constructs regression models based on Bayesian learning techniques. This assessment has been validated in a number of studies and has been shown to be highly predictive of RNA quality (Mueller et al., 2004; Imbeaud et al., 2005; Schroeder et al., 2006). It was found that all samples with 260/280 ratios between 1.8 and 2.2 (considered optimal) had RIN of between 7 and 10 Moreover, samples that did not have a ratio of between 1.8 and 2.2 had a RIN lower than 7. This provides further evidence that this RNA is of high quality and suitable for RT-qPCR and/or RNA seq analysis.

**FIGURE 3:**
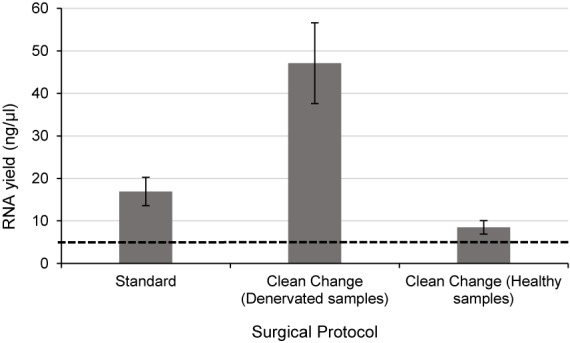
Even when the time delay is minimized/equivalent between samples there is still a large differential In RNA yield due to exposure to surgical antiseptics. Denervated samples were liberated under a standard (*n* = 4) and “clean change” (*n* = 3) surgical protocol. Samples liberated in a surgical environment where the “clean change” surgical protocol was implemented yielded RNA concentrations significantly higher than those liberated under standard conditions (*p* < 0.01, two tailed *t*-test). Healthy nerve samples were also liberated under a “clean change” protocol (*n* = 3) which yielded significantly lower concentrations of RNA compared to denervated samples < *p* < 0.001 two tailed *t*-test). The dotted black line represents the minimum concentration of RNA often required for downstream RT-qPCR/RNA seq. Data are means ± 1 SD.

Since RNA yields from human denervated tissue remained lower than that reported from rodent studies (Yamamoto et al., 2012; Weng et al., 2018), this necessitated further exploration of peri-operative variables. Specifically, the effect of surgical antiseptics on RNA yield. Figure 3 suggests that samples liberated using the “clean change” surgical protocol (Group 3) yielded RNA in concentrations 2.8 times higher than those extracted under standard conditions (*p* < 0.01) (Groups 1 and 2). In comparing denervated tissue to healthy nerve samples, the concentration of RNA isolated from healthy nerve samples was significantly lower than that from denervated tissue (*p* < 0.05) (Figure 3). Assessments of RNA quality (260/280 absorbance ratios, ratio of ribosomal RNA bands in agarose gels and changes in electropherogram morphology) suggested that the RNA was of high quality (Figure 2) similar to the quality of RNA from nerve samples cryopreserved within 3 min (Figure 2).

**FIGURE 4:**
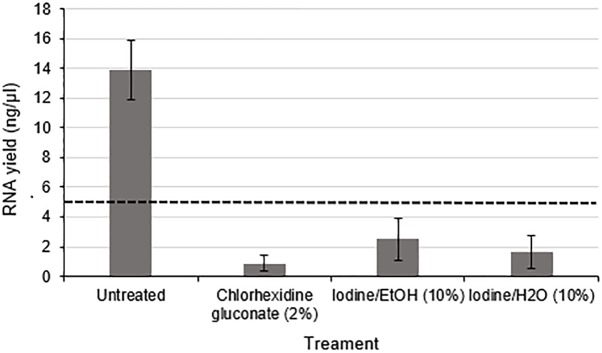
RNA yield from rodent nerves is reduced following exposure to surgical antiseptic reagents There was a statistically significant difference between the untreated (*n* = 8) and each of the treated groups (2% Chlorhexidine gluconate (*n* = 6), 10% lodine/EtOH (*n* = 6). 10% lodine/H2O < *n* = 6) as assessed by a one way ANOVA and Dunnett’s test (*p* < 0.0l between each treatment group and the untreated group). Data are means ± 1 SD. The black dotted line at 5 ng/ul represents the minimum concentration of RNA required for downstream RT-qPCR and RNA seq applications.

Using nerve tissue freshly harvested from rats under carefully controlled environmental conditions enabled the effects of antiseptic reagents to be studied in isolation. A significant decrease in the yield of RNA (approximately 8.3 fold lower in exposed nerves compared to the untreated group) (*p* < 0.01) was detected following exposure of rodent nerve samples to each of the different antiseptic reagents (Figure 4).

## Discussion

In order to establish a protocol for the reliable extraction of RNA from human nerve samples, this study set out to characterize peri-operative variables predictive of RNA yield. The effect on RNA yield of time delays between liberation of the nerve sample and snap freezing was investigated and results suggested that nerve samples should be snap frozen within 3 min to preserve RNA quantity and quality. This time interval is considerably shorter than that cited in other studies that have extracted RNA from surgical specimens which have shown that time delays of several hours between surgical liberation of a sample and cryopreservation is detrimental to RNA quantity and quality (Mee et al., 2011; Samadani et al., 2015; Patel et al., 2017). Studies of surgically harvested tissue have largely been limited to the study of non-fibrous cancerous tissues such as breast and prostate (Mee et al., 2011; Samadani et al., 2015; Patel et al., 2017). These studies achieved optimal yields of RNA (sufficient for RT-qPCR and RNA seq analysis) largely through the optimisaiton of RNA extraction protocols alone. This is perhaps attributable to the fact that the cancerous tissues explored in these studies have higher cellular densities than nerve tissue and thus more RNA that can be isolated, perhaps diminishing the impact of time delays and/or exposure of samples to antiseptic reagents on the quantity and quality of isolated RNA. Another major difference between nerve samples and other organs is that the nerve trunk contains bundles of axons, together with Schwann cells, fibroblasts, endothelial cells, perineurial cells and other associated cells, but the cell bodies of the neurons are not present since they are located within the CNS or adjacent ganglia. Therefore the RNA which is obtained from excised nerve samples will be predominantly derived from Schwann cells and other non-neuronal cells rather than neurons.

Even when delay was minimized, RNA yields from human nerves in this study remained lower than those reported in rodent studies of denervated nerve tissue (Yamamoto et al., 2012; Weng et al., 2018). Therefore the exploration of other peri-operative variables such as the interaction of samples with antiseptic surgical reagents such as chlorhexidine and iodine was necessitated. This study showed for the first time that exposure of nerves to surgical antiseptic reagents had detrimental effects on the quantity of RNA that was isolated from the samples. Chlorhexidine and povidone-iodine based antiseptic reagents are cytotoxic to prokaryotic and eukaryotic cells. Chlorhexidine works by binding to the cell membrane causing it to rupture. On the other hand, povidone-iodine has a broader spectrum of antimicrobial activity. It works by crossing the cell membrane and destroying microbial proteins as well as DNA. It follows that these reagents may have acute cytotoxic effects within the nerve samples thus impairing RNA yield.

It was evident that healthy nerve samples yielded significantly lower quantities of RNA than that from denervated tissue, which concurs with rodent studies (Yamamoto et al., 2012; Weng et al., 2018) even when the exposure of nerve samples to antiseptic reagents was minimized and the time delay was limited to 3 min. The differential observed in both species is likely to be due to the biological mechanisms that underpin nerve regeneration. Evidence from rodent and human models of nerve injury have shown that Wallerian degeneration starts soon after nerve injury, involving a complex cascade of events including degradation of the fibrous connective tissue (Rotshenker, 2011). This may make denervated tissue more amenable to lysis and cellular disruption leading to higher yields of RNA compared with intact healthy nerve tissue. Furthermore, Wallerian degeneration involves proliferation of Schwann cells as well as infiltration and proliferation of other non-neuronal cells (such as macrophages and other immune system cells) (Rotshenker, 2011), potentially contributing to increased RNA content in denervated nerve tissue. This finding highlights the pertinence of considering the innervation status of nerve tissue in order to optimize RNA yield.

In order to isolate and further investigate the effects of exposure to antiseptic reagents, this study used a rodent model of peripheral nerve liberation which showed that these antiseptic reagents reduced RNA yields significantly. Chlorhexidine and iodine based reagents can be found in abundance in operating theaters around the world where they are often deployed for preoperative skin preparation. This finding, together with experimental evidence that has shown iodine and chlorhexidine based reagents to have cytotoxic effects on *in vitro* populations of human neuronal cells and rodent Schwann cells (Doan et al., 2012), necessitates further work to characterize the effect of these reagents on the regenerative capacity of the peripheral nervous system. This could potentially inform the modification and development of surgical tissue handling protocols more generally, beyond just the focus here on obtaining nerve tissue RNA for research.

In addition to influencing RNA extraction, iodine and chlorhexidine based reagents may have downstream effects on qPCR assays. These reagents have been shown to inactivate the Human Immunodeficiency Virus through a mechanism thought to be at least partially attributable to the ability of these reagents to manipulate the viral DNA reverse transcriptase (Harbison and Hammer, 1989; Sattar and Springthorpe, 1991). This enzyme is analogous to the RNA to cDNA reverse transcriptase step used in RT-qPCR and RNA seq assays, providing an additional reason to avoid the contamination of samples intended for downstream qPCR and RNA seq assays.

In summary, this study reports new experimental evidence from human and animal studies that reveals the effects of time delays and surgical antiseptics on the RNA yield obtained from nerve tissue. This information can help to inform the development of improved methodology, specifically limiting time delay between sample liberation and cryopreservation to less than 3 min whilst utilizing a “clean change” surgical protocol to reduce antiseptic exposure. These findings provide new information about the response of fresh nerve tissue following isolation, including differences between healthy and denervated samples. This understanding will enable more effective use to be made of valuable human nerve tissue samples, addressing the knowledge gaps that currently exist in studying cellular and molecular mechanisms that underpin human nerve regeneration.

## Ethics Statement

This study was carried out in accordance with the recommendations of ‘HTA guidelines, Biobank Ethical Review Committee’ with written informed consent from all subjects. All subjects gave written informed consent in accordance with the Declaration of Helsinki. The protocol was approved by the UCL Biobank Ethical Review Committee.

## Author Contributions

MW designed the concept and experimental methods used to assess quality and quantity of RNA yields, executed the experiments, performed analysis, and wrote the manuscript. TQ contributed to experimental design and clinical data detailed in the manuscript, made comments on the manuscript, and involved in writing up. JP contributed to experimental design and data analysis and informed the writing of the manuscript.

## Conflict of Interest Statement

The authors declare that the research was conducted in the absence of any commercial or financial relationships that could be construed as a potential conflict of interest. The handling Editor is currently co-organizing a Research Topic with one of the authors JP, and confirms the absence of any other collaboration.
